# Evaluation of salivary biomarkers for the diagnosis of periodontitis

**DOI:** 10.1186/s12903-021-01600-5

**Published:** 2021-05-17

**Authors:** Yong Zhang, Ni Kang, Fei Xue, Jing Qiao, Jinyu Duan, Fan Chen, Yu Cai

**Affiliations:** 1 Department of First Clinical Division, Peking University School and Hospital of Stomatology and National Center of Stomatology and National Clinical Research Center for Oral Diseases and National Engineering Laboratory for Digital and Material Technology of Stomatology and Beijing Key Laboratory of Digital Stomatology, Beijing, 100081 People’s Republic of China; 2 Department of Periodontology, Peking University School and Hospital of Stomatology and National Center of Stomatology and National Clinical Research Center for Oral Diseases and National Engineering Laboratory for Digital and Material Technology of Stomatology and Beijing Key Laboratory of Digital Stomatology, No.22 South Avenue Zhongguancun, Beijing, 100081 People’s Republic of China; 3 Central Laboratory, Peking University School and Hospital of Stomatology and National Center of Stomatology and National Clinical Research Center for Oral Diseases and National Engineering Laboratory for Digital and Material Technology of Stomatology and Beijing Key Laboratory of Digital Stomatology, Beijing, 100081 People’s Republic of China; 4 Department of Stomatology, People’s Hospital of Peking University, No.11 Beijing Xizhimen South Street, Beijing, 100044 People’s Republic of China

**Keywords:** Saliva, Biomarker, Diagnosis, Periodontitis, Gingivitis

## Abstract

**Background:**

Salivary interleukin (IL)-1β, matrix metalloproteinase (MMP)-8, pyridinoline cross-linked carboxyterminal telopeptide of type I collagen (ICTP) and *Porphyromonas gingivalis* (Pg) are related to periodontitis. This study aimed to investigate the diagnostic potential of these biomarkers and to build a prediction panel for diagnosing periodontal disease.

**Methods:**

A total of 80 participants were enrolled in a cross-sectional study and divided into healthy (n = 25), gingivitis (n = 24), and periodontitis (n = 31) groups based on their periodontal exam results. A full mouth periodontal examination was performed and unstimulated saliva was collected. Salivary IL-1β, MMP-8, ICTP, and Pg were assessed using enzyme-linked immunosorbent assay (ELISA) and quantitative real time PCR (qPCR). Their potentials for diagnosing periodontal disease were analyzed and combined prediction panels of periodontal disease were evaluated.

**Results:**

As a single marker, IL-1β showed the best diagnostic value of the four markers evaluated and exhibited an area under the curve (AUC) value of 0.88 with 90% sensitivity and 76% specificity for discriminating periodontitis subjects from healthy subjects, an AUC value of 0.80 with 83% sensitivity and 76% specificity for discriminating gingivitis subjects from healthy subjects and an AUC value of 0.66 with 68% sensitivity and 64% specificity for differentiating periodontitis subjects from gingivitis subjects. The combination of IL-1β, ICTP, and Pg exhibited the highest efficacy for discriminating periodontitis subjects from healthy subjects (AUC = 0.94) and gingivitis subjects (AUC = 0.77). The combination of IL-1β and MMP-8 exhibited the best ability to discriminate gingivitis from healthy subjects (AUC = 0.84).

**Conclusions:**

Salivary IL-1β, MMP-8, ICTP, and Pg showed significant effectiveness for diagnosing periodontal disease. The combination of IL-1β, ICTP, and Pg can be used to discriminate periodontitis subjects from healthy subjects and gingivitis subjects, and the combination of IL-1β and MMP-8 can be used to discriminate gingivitis subjects from healthy subjects.

## Background

Periodontal disease is a chronic inflammatory disease induced by pathogenic bacteria that results in connective tissue and alveolar bone destruction [1]. Traditionally, diagnosis of periodontal disease has been based on clinical and radiographic examinations that reflect a previous history of disease but lack ability to detect current disease activity [2]. Early detection of periodontal tissue destruction can be useful to monitor a disease’s progression and prevent future destruction. Saliva collection is a simple noninvasive bodily fluid test that can be effective for diagnosis, because saliva contains omics constituents that can reflect the current physiological status of periodontal tissue [3, 4].

In the past several decades, various salivary markers (bacteria [5], host enzymes [6], cytokines [7], and bone metabolic products [8]) have been investigated as targets to differentiate between periodontitis patients and healthy subjects. However, there have been inconsistent or even contrary results in previous studies [9–11]. As we know, during the progress of periodontal disease, in the gingivitis stage, patients exhibit gingival inflammation without connective tissue or bone destruction. In the early stage of periodontitis, patients display gingival inflammation with connective tissue destruction and progress to alveolar bone destruction at later stages of disease. We believe that the change of markers occurs consecutively at different phases of periodontal disease, so a biomarker combination can be more effectively used for diagnosing disease status. Salivary interleukin (IL)-1β [12], matrix metalloproteinase (MMP)-8 [13], pyridinoline cross-linked carboxyterminal telopeptide of type I collagen (ICTP) [14], and *Porphyromonas gingivalis* (Pg) [15] display high prevalence in the populations of interest and are strongly related to periodontitis. The aim of this study is to evaluate the diagnostic efficiency of these markers (representing inflammation, tissue degradation, and periodontal pathogens) among healthy, gingivitis, and periodontitis subjects and to combine them in order to build an effective prediction panel for diagnosing periodontal disease.

## Methods

### Study design

This study was designed and performed as a cross-sectional study. It was approved by the human subjects ethics board of Peking University School and Hospital of Stomatology and was conducted in accordance with the Helsinki Declaration of 1975, as revised in 2013. All participants were informed verbally and in writing and each provided written informed consent. All primary data were collected according to Strengthening the Reporting of Observational Studies in Epidemiology guidelines.

The inclusion criteria were: (1) 20–65 years of age, (2) having at least 20 teeth, (3) underwent no medical treatment during the last 3 months before examination and sampling (4) non-smoker, (5) no history of systemic disease.

The exclusion criteria were (1) wore orthodontic appliances, (2) pregnant or currently in the breast-feeding period, (3) having undergone periodontal therapy within the 6 months prior to the examination and sampling.

### Clinical evaluations

All participants were recruited at the department of periodontology, first clinical division, Peking University School and Hospital of Stomatology. All participants received a full mouth periodontal examination and a medical and dental history evaluation by one single examiner. All permanent teeth were measured with a 10-mm manual periodontal probe (PCP10-SE, Hu-Friedy, Chicago, USA) and measurements were rounded upwards to the nearest millimeter. Plaque Index (PI) and bleeding on probing (BOP) were measured at 4 sites (mesial, distal, buccal, and lingual). Probing depth (PD) and clinical attachment loss (CAL) were measured at 6 sites on all teeth. BOP was considered present if the probed site bled for approximately 20 s after probing.

### Patient groups

Based on the results of the examinations, all participants were divided into 3 groups: healthy group (H), gingivitis group (G) and periodontitis group (P) in accordance with the 2017 World Workshop on the Classification of Periodontal and Peri-Implant Diseases and Conditions [16, 17].

1. Healthy group (H): Subjects with absence of bleeding on probing (BOP < 10%), probing depth (PD) ≤ 3 mm, no clinical attachment loss (CAL), no radiographic bone loss, no sign of other inflammatory lesions in the oral mucosa.
2. Gingivitis group (G): Subjects with presence of bleeding on probing and BOP ≥ 10%, PD ≤ 3 mm, no clinical attachment loss, no radiographic bone loss.
3. Periodontitis group (P): Subjects with presence of interdental CAL ≥ 5 mm, PD ≥ 6 mm and radiographic bone loss extending to 2/3 of the root or beyond. Participants had lost no more than 4 teeth due to periodontitis. In addition, to estimate periodontitis progression, disease stage and grade was determined by evaluating radiographic bone loss/age [18]. Radiographic bone loss was determined using the tooth showing the most severe bone loss as a percentage of root length. Since the values of % bone loss/age were > 1.0. All participants were considered to be in stage III grade C periodontitis.

### Saliva collection and analysis

Saliva collection was performed according to the technique proposed by Henson et al. [19]. Participants were asked to refrain from eating, drinking, smoking or engaging in oral hygiene procedures for at least two hours prior to saliva collection. Subjects rinsed their mouths with tap water for 30 s approximately 10 min prior to saliva collection and then expectorated into sterile tubes while seated in an upright position. 5 ml of unstimulated saliva samples were collected, then saliva samples were centrifuged at 5000 g for 5 min at 4 °C. Supernatants were removed from the pellet. 0.5 ml aliquots of the resultant supernatant and pellets were stored at − 80 °C until analysis.

Salivary IL-1β, MMP-8, and ICTP levels were detected and measured using commercial enzyme-linked immunosorbent assay (ELISA) kits obtained from R&D Systems (Minneapolis, MN, USA) and Orion Diagnostica (Espoo, Finland), according to the manufacturer’s instructions. Salivary Pg DNA was extracted from pellets using UltraClean Microbial DNA Isolation Kits (MO BIO Laboratories Inc, Carlsbad, California, USA)Then, quantities of Pg were determined using quantitative real time PCR (qPCR). Primers for the 16S rRNA gene of Pg were used as follows: forward primer, 5′-GCGCTCAACGTTCAGCC-3′; reverse primer, 5′-CACGAATTCCGCCTGC-3′. qPCR was performed in duplicate in reaction volumes of 10 μl using Power SYBR-Green Master Mix (Applied Biosystems, Foster City, California, USA) for 15 min at 95.8 °C for initial denaturing, followed by 40 cycles of 95.8 °C for 30 s and 60.8 °C for 30 s. Cycle Threshold (Ct) values were calculated subsequent to this procedure.

### Statistical analysis

Based on the results from Wu et al. [20], Mishra et al. [14], and Zeller et al. [21] that compared IL-1β, MMP-8, ICTP, and Pg between periodontitis patients and healthy subjects, we assumed an equal standard deviation in the healthy, gingivitis, and periodontitis groups. G*Power 3.1.9.2 software was used to perform sample calculations, using F-test for one-way ANOVA, considering effect size of 0.4, statistical power of 80%, significance level of 95% (α < 0.05) two-tailed. Based on this, a minimum of 22 participants were required for each group to indicate a difference between groups and this was set as the sample size requirement of the study. The SPSS statistical program (Version 21.0; SPSS Inc., Chicago, IL, USA) was used to analyze the data. The mean with standard deviation was used to describe the variables of the demographic and clinical characteristics as well as the distribution of biomarkers among the healthy, gingivitis, and periodontitis groups. Independent t-tests were used to evaluate the differences between the three groups. Correlations between biomarkers and clinical indications were assessed using a linear regression analysis and logistic regression adjusted for age and gender. These results were used to establish panels for predicting gingivitis and periodontitis according to an automatic stepwise selection strategy. Receiver operating characteristic (ROC) curve analysis and corresponding area under the curve (AUC) analyses were used to evaluate the performance of biomarkers and predictive panels. Cut-off values were obtained using the ROC curves. The sensitivity and specificity for the biomarker combinations were estimated by identifying the cut-off point of the predicted probability that yielded the highest sum of sensitivity and specificity. Statistical significance was defined as *p* < 0.05.

## Results

A total of 80 participants were enrolled in this study. The demographic and clinical characteristics of all participants are presented in Table 1. Gender demographics and tooth loss status were balanced among the three groups. 25 patients were placed into the healthy group (H), 24 patients were placed into the gingivitis group (G) and 31 patients were placed into the periodontitis group (P). As anticipated, there were no significant differences found in the salivary flow rates between these three groups. Compared to the healthy group, the diseased (gingivitis and periodontitis) groups showed higher PI and BOP. The periodontitis group showed older, higher PD and CAL compared to healthy and gingivitis groups.

**Table 1:** Characteristics of study participants between the healthy, gingivitis, and periodontitis groups

	Healthy group (N = 25)	Gingivitis group(N = 24)	Periodontitis group(N = 31)
Age	24.68 ± 3.52	26.32 ± 4.02	42.58 ± 3.39 ** ##
Gender (M/F)	12/13	11/13	17/14
Tooth	27.7 ± 1.1	27.6 ± 1.2	27.5 ± 0.9
Plaque index (PI)	0.22 ± 0.28	0.76 ± 0.58 *	1.32 ± 0.76 *, #
Bleeding on probing (BOP, %)	1.1 ± 1.8	25.8 ± 9.7 **	65.1 ± 17.1 ** ##
Probing depth (PD, mm)	2.62 ± 0.45	2.95 ± 0.29 **	4.74 ± 0.64 ** ##
Clinical attachment loss (CAL, mm)	0	0	4.93 ± 0.52 ** ##
Unstimulated salivary flow rates (ml/min)	0.58 ± 0.19	0.54 ± 0.15	0.56 ± 0.14
IL-1β (pg/ml)	92.2 ± 31.9	128.6 ± 33.5 **	162.2 ± 55.9 ** #
MMP-8 (ng/ml)	435.8 ± 180.6	603.2 ± 220.7 *	657.1 ± 279.8 **
ICTP (pg/ml)	528.8 ± 141.1	598.0 ± 203.2	789.7 ± 246.8 ** ##
Pg (Ct value)	13.78 ± 1.23	14.52 ± 1.01 *	15.20 ± 1.07 ** #

*Significantly different compared to healthy group (*p < 0.05, **p < 0.01)

#Significantly different compared to gingivitis group (#*p* < 0.05, ##*p* < 0.01)

The salivary level of IL-1β, MMP-8, ICTP, and Pg were measured and are shown in Table 1. ANOVA analysis was performed to investigate difference between the three groups. The periodontitis group showed significantly higher levels of IL-1β, MMP-8, ICTP, and Pg compared to the healthy group and higher levels of IL-1β, ICTP, and Pg compared to the gingivitis group. The gingivitis group showed higher levels of IL-1β, MMP-8, and Pg compared to the healthy group. No significant difference in MMP-8 was found between the periodontitis and gingivitis groups; no significant difference in ICTP was found between the gingivitis and healthy groups.

We next evaluated the correlation between each marker and clinical indices (Age, PD, and BOP) using a linear regression model. The results showed that IL-1β and ICTP were moderately correlated with PD (r = 0.43, 0.46, *p* < 0.01) and BOP (r = 0.43, 0.48, *p* < 0.01); Pg was moderately correlated with PD (r = 0.41, *p* < 0.01) and mildly correlated with BOP (r = 0.38, *p* < 0.01); MMP-8 was mildly correlated with PD (r = 0.32, *p* < 0.01) and BOP (r = 0.38, *p* < 0.01) (Table 2). None of these four markers showed significant correlation with age, and after employing the Mann–Whitney U-test, no gender-specific differences in the levels of these markers were found (*p* > 0.05).

1. Periodontitis group versus healthy group

**Table 2:** Correlation analysis between markers and clinical characteristics of study population

	PD		BOP		Age	
	Spearman’s ρ	*p* value	Spearman’s ρ	*p* value	Spearman’s ρ	*p* value
IL-1β	0.428	0.001	0.433	< 0.001	0.107	0.560
MMP-8	0.319	0.008	0.377	0.002	0.161	0.187
ICTP	0.458	< 0.001	0.479	< 0.001	0.167	0.155
Pg	0.414	0.004	0.376	0.002	0.072	0.274

**Fig. 1:**
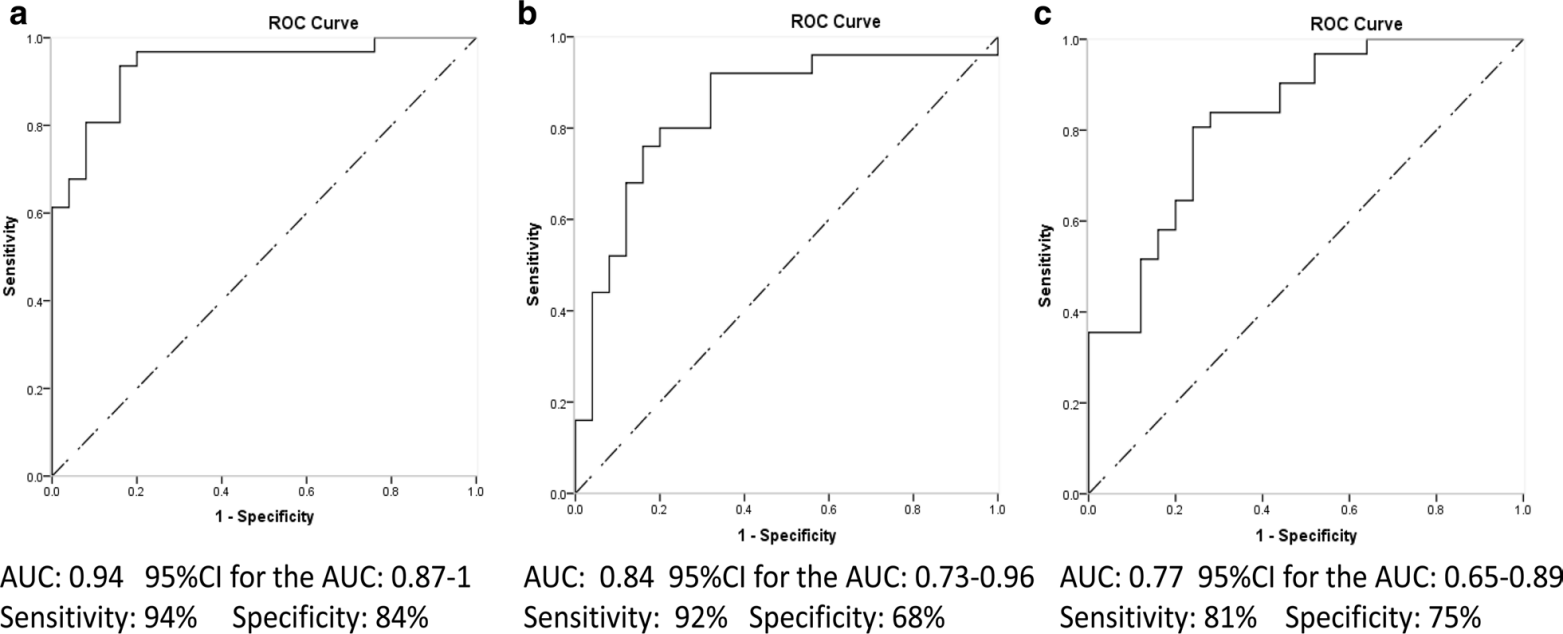
Receiver operating characteristic (ROC) analysis of salivary biomarker combinations with AUC values: **a** The combination of IL-1β, ICTP, and Pg used to discriminate periodontitis patients from healthy subjects. **b** The combination of IL-1β and MMP-8 used to discriminate gingivitis patients from healthy subjects. **c** The combination of IL-1β, ICTP, and Pg used to discriminate periodontitis patients from gingivitis patients

All four markers tested in this study showed a significant difference between the periodontitis and healthy groups (Table 1). IL-1β yielded an AUC value of 0.88, and the other three markers yielded AUC values ranging from 0.75 to 0.85 (Table 3). Logistic regression was used to evaluate different combinations of the four biomarkers. Among the different combinations, IL-1β combined with ICTP yielded a higher AUC value of 0.92 with 87% sensitivity and 80% specificity. The combination of IL-1β, ICTP, and MMP-8 yielded an AUC value of 0.93 with 90% sensitivity and 84% specificity. The combination of IL-1β, ICTP, and Pg yielded an AUC value of 0.94 with 94% sensitivity and 84% specificity (Fig. 1). All four biomarkers combined yielded the best AUC value of 0.95 (Table 4).

2. Gingivitis group versus healthy group

IL-1β, MMP-8, and Pg levels were shown to be significantly different between the gingivitis group and the healthy group (Table 1). IL-1β and MMP-8 yielded similar AUC values, Pg yielded a lower AUC value (Table 3). Logistic regression analysis was used to identify differences among these biomarker combinations. IL-1β and MMP-8 in combination yielded an AUC value of 0.84 with 92% sensitivity and 68% specificity (Fig. 1). The combination of IL-1β, MMP-8, and Pg yielded a better AUC value of 0.85, with 83% sensitivity and 80% specificity (Table 5).

3. Periodontitis group versus gingivitis group

**Table 3:** Diagnostic potential of salivary biomarkers between periodontitis patients and healthy subjects

Biomarker	Cut-off value	AUC value	95% CI	*p* value	Sensitivity (%)	Specificity (%)	Positive predictive (%)	Negative predictive (%)
Periodontitis versus Healthy (P versus H)								
IL-1β (pg/ml)	91.1	0.875	0.785–0.965	< 0.001	90.3	76.0	82.4	86.4
MMP-8 (ng/ml)	594.6	0.761	0.630–0.892	0.001	67.7	88.0	87.5	68.8
ICTP (pg/ml)	625.0	0.840	0.727–9.953	< 0.001	80.6	88.0	89.3	78.6
Pg (Ct value)	13.81	0.788	0.668–0.908	< 0.001	87.1	56.0	71.1	77.8
Gingivitis versus Healthy (G versus H)								
IL-1β (pg/ml)	104.6	0.803	0.671–0.935	< 0.001	83.3	76.0	76.9	82.6
MMP-8 (ng/ml)	583.6	0.766	0.630–0.903	0.001	66.7	84.0	80.0	72.4
ICTP (pg/ml)	585.0	0.594	0.435–0.754	0.252	62.5	64.0	62.5	64.0
Pg (Ct value)	14.17	0.687	0.534–0.840	0.023	75.0	64.0	66.7	72.7
Peridoontitis versus Gingivitis (P versus G)								
IL-1β (pg/ml)	147.6	0.657	0.490–0.785	0.029	67.7	64.0	70.0	61.5
MMP-8 (ng/ml)	670.7	0.563	0.412–0.714	0.419	58.1	52.0	60.0	50.0
ICTP (pg/ml)	676.0	0.707	0.612–0.783	0.002	77.4	72.0	77.4	72.0
Pg (Ct value)	14.88	0.698	0.555–0.841	0.011	67.7	76.0	77.8	65.5

**Table 4:** Performance of salivary biomarker combinations to discriminate periodontitis patients from healthy subjects

	Sensitivity (%)	Specificity (%)	AUC value	95% CI
IL-1β + MMP-8	87.1	80.0	0.883	0.793–0.972
IL-1β + Pg	87.1	84.0	0.910	0.828–0.991
IL-1β + MMP-8 + Pg	90.3	84.0	0.920	0.843–0.997
ICTP + MMP-8	83.9	76.0	0.850	0.746–0.955
ICTP + Pg	83.9	80.0	0.881	0.794–0.969
IL-1β + ICTP	87.1	80.0	0.917	0.840–0.995
IL-1β + ICTP + MMP-8	90.3	84.0	0.929	0.865–0.993
IL-1β + ICTP + Pg	93.5	84.0	0.935	0.873–0.998
IL-1β + ICTP + MMP-8 + Pg	93.5	84.0	0.946	0.892–0.999

**Table 5:** Performance of salivary biomarker combinations to discriminate gingivitis patients from healthy subjects

	Sensitivity (%)	Specificity (%)	AUC value	95% CI
IL-1β + MMP-8	91.6	68.0	0.842	0.726–0.957
IL-1β + Pg	79.2	76.0	0.819	0.699–0.940
IL-1β + MMP-8 + Pg	83.3	80.0	0.853	0.743–0.962
IL-1β + MMP-8 + Pg + ICTP	87.5	76.0	0.850	0.740–0.965

IL-1β, ICPT, and Pg levels were significantly different between the periodontitis and gingivitis groups and yielded similar AUC values (Tables 1, 3). After logistic regression analysis, among different combinations of two markers, the combination of IL-1β and ICTP yielded an AUC value of 0.76 with 81% sensitivity and 71% specificity, the combination of IL-1β and Pg yielded a similar AUC value, and the combination of IL-1β, Pg, and ICTP yielded the best AUC value of 0.77 with 81% sensitivity and 75% specificity (Table 6, Fig. 1).

**Table 6:** Performance of salivary biomarker combinations to discriminate periodontitis from gingivitis

	Sensitivity (%)	Specificity (%)	AUC value	95% CI
IL-1β + ICTP	80.6	70.8	0.755	0.621–0.889
IL-1β + Pg	77.4	70.8	0.720	0.584–0.856
ICTP + Pg	74.2	75.0	0.747	0.618–0.876
IL-1β + ICTP + Pg	80.6	75.0	0.770	0.647–0.894
IL-1β + ICTP + MMP-8 + Pg	83.9	70.8	0.770	0.647–0.896

## Discussion

Periodontal disease has historically been diagnosed using a patient’s clinical performance in BOP, PD, and CAL tests as well as radiographic evidence of alveolar bone loss. These methods are reliable, but are also costly and depend upon a clinician's professional experience [2]. Saliva has been proven to be a tool with a high potential value for early diagnosis and monitoring oral and systemic diseases [4, 22]. A number of salivary markers have been demonstrated to be significantly different between diseased and healthy subjects, but up to now there has been no clear and convincing biomarker that can be used for diagnosing periodontal disease. The present study proposes efficient salivary panels for diagnosing gingivitis and periodontitis and the development of saliva-based point of care (POC) technology tools that could be used in chair-side diagnostics, self-screenings, and risk-assessment [23].

We need a balance between microbial and host response to maintain periodontal health during the progress of periodontal disease. If the balance is broken, bacterial invasion, host inflammatory response, tissue and bone destructions occur non-simultaneously. After bacterial (Pg) invasion, markers of inflammation (IL-1β) are released [24]. Enzymes such as MMP-8 are produced and activated by host cells leading to the degradation of connective tissue [25], and bone degradation results in the release of ICTP into periodontal tissues and saliva [8]. Kuula’s [26] and Hamedi’s [27] studies revealed positive correlations between Pg infection and IL-1β or MMP-8 levels. Therefore, we selected these four marker candidates (IL-1β, MMP-8, ICTP, and Pg) and evaluated their efficiency for diagnosing gingivitis and periodontitis.

IL-1β is a well-known inflammatory stimulator that can be used to discriminate between healthy and periodontal lesions [7]. Pg is also significantly associated with periodontal disease and has been used as a potential screening biomarker of periodontitis [28]. In this study, both of IL-1β and Pg showed significantly different levels among the three subject groups. Their salivary levels increased in the gingivitis group and were higher still in the periodontitis group. Our results were consistent with other studies [15, 29]. Besides significant elevation of these biomarkers in the gingivitis and periodontitis groups, our results revealed positive correlation between IL-1β, Pg, and clinical indices (PD and BOP). IL-1β and Pg indeed reflected periodontal status and may be valuable targets for predicting periodontal disease.

Recent studies have shown that MMP-8 is an indicator for early periodontitis in particular [23, 30, 31]. In our study, MMP-8 was detected in significantly higher levels within the diseased groups (gingivitis and periodontitis) compared to the healthy group. However, there was no significant difference between their levels in the periodontitis and gingivitis groups. This result is in accordance with Morelli’s [32] and Nascimento’s [33] results. Verhulst [34] has also reported that MMP-8 is not associated with periodontitis. However in Heikkinen’s [31] and Yucel’s [35] studies, salivary MMP-8 was significantly elevated in periodontitis patients compared to gingivitis patients, and in Syndergaard’s [10], Yucel’s [35], and Noack’s [36] studies, there were no significant difference in MMP-8 between gingivitis groups and healthy groups. These inconsistent results may be due to the evaluation of total MMP-8 in our study and Verhulst’s [34] study. Sorsa’s study [37] and others [38, 39] have demonstrated that total MMP-8 may not be able to effectively detect periodontal breakdown or progression of periodontitis and they concluded that, instead of total MMP-8, active MMP-8 (aMMP-8) levels truly reflect a proinflammatory state of periodontal disease. Assessment of aMMP-8 may have shown a direct correlation with periodontal disease.

Before an effective diagnosis of periodontitis, a considerable amount of alveolar bone destruction must be established. When we measured the bone loss clinically, a 2–3 mm threshold change was needed before exhibiting obvious destruction. This may delay diagnosis and treatment [40]. As a breakdown product of Type I collagen, ICTP is the major constituent of alveolar bone and is considered to reflect alveolar bone degradation and periodontal disease activity [41]. In our study, ICTP was not found to be significantly different between the gingivitis group and the healthy group, because there was no bone loss in these two groups. Apparent alveolar bone loss in the periodontitis group resulted in significantly higher ICTP levels compared to the gingivitis and healthy groups. This is in accordance with Mishra’s [14] and Giannobile’s [42] studies where they concluded that increased ICTP can be used to differentiate active gingivitis from periodontitis. Payne's [43] results have also stated that salivary ICTP concentration was significantly correlated with alveolar bone loss.

After confirming the differing biomarker results found between the periodontitis, gingivitis, and healthy groups, we examined their ability to discriminate between different periodontal clinical phenotypes. As a single marker, IL-1β showed the best diagnostic value of these four candidates; it exhibited an AUC value of 0.88 with 90% sensitivity and 76% specificity for discriminating periodontitis subjects from healthy subjects, an AUC value of 0.80 with 83% sensitivity and 76% specificity for discriminating gingivitis subjects from healthy subjects, and an AUC value of 0.66 with 68% sensitivity and 64% specificity for discriminating periodontitis subjects from gingivitis subjects. These are valuable results and are consistent with results from Jaedicke’s [7] and Nazar’s [44] investigations. These both concluded that IL-1β is the most robust salivary biomarker with respect to periodontal disease. Hassan’s [45] results also exhibited a positive relationship between salivary IL-1β and gingival inflammation during pregnancy. All of these results support our inclusion of IL-1β as a predictive overall indicator of gingivitis and periodontitis.

Different markers may peaked at different stages over the course of disease, and when biomarkers of host and microbial origin are combined, the detection of periodontitis maybe be improved [20, 46, 47]. Previous studies have pointed towards realizing a stronger discriminatory capability when IL-1β, MMP-8, and other markers are combined, compared to single-marker analysis [48]. Pg and MMP-8 in combination [49], as well as ICTP and MMP-8 in combination [50], have also exhibited greater predictive value. Our data show that IL-1β, individually, revealed an AUC value of 0.88 for discriminating periodontitis subjects from healthy subjects. The combination of IL-1β, MMP-8, and Pg strongly improved this performance to an AUC value of 0.92. This is consistent with Gursoy's [51, 52] results. In that study, IL-1β, MMP-8, and Pg were calculated together to obtain a cumulative risk score that was highly correlated with advanced periodontitis. Although this previous study showed that biomarker combinations facilitate a more robust prediction of periodontal progression and stability, our results were different from Gursoy’s [51, 52]. We demonstrated that IL-1β and ICTP in combination yielded a similar AUC value (0.917) to differentiate periodontitis subjects from healthy subjects when compared to the combination of IL-1β, MMP-8, and Pg (0.920), indicating that IL-1β and ICTP are more effective for predicting periodontitis. The combination of IL-1β, ICTP, and Pg exhibited the best AUC value (0.94) to discriminate periodontitis subjects from healthy subjects.

As a nondestructive and reversible gingival inflammation stage, we enrolled gingivitis subjects into this study and assigned participants with more homogeneous clinical phenotypes. Our results showed that MMP-8 was not significantly elevated in the periodontitis group compared to the gingivitis group, and ICTP was not significantly elevated in the gingivitis group compared to the healthy group. This indicates that, for predicting the disease status, different marker combinations should be used to achieve an effective diagnosis. After logistic regression analysis, the combination of IL-1β, ICTP, and Pg not only yielded the best AUC value to discriminate periodontitis patients from healthy subjects, but also exhibited the best ability to discriminate periodontitis subjects from gingivitis subjects (AUC = 0.77). To discriminate gingivitis from healthy subjects, IL-1β, MMP-8, and Pg together exhibited the best AUC value of 0.85, while IL-1β and MMP-8 in combination yielded a slight lower AUC value of 0.84. These AUC values were lower than the combination most effective at discriminating periodontitis subjects from healthy subjects (IL-1β, ICTP and Pg, with an AUC of 0.94), but were above acceptable AUC values of 0.75 [53] and can potentially be used for clinical diagnosis.

However, there were some limitations in this study. We directly measured salivary concentrations of these markers, while Afacan’s [54] study suggests that salivary flow rate may result in differences in saliva composition. Calibrated-total-protein salivary biomarker levels may be more effective to evaluate the diagnostic power of these biomarkers. We also evaluated the qPCR levels of total Pg in saliva and other studies have shown that P. *gingivalis* can be divided according to fimA genotypes; fimA type I is exclusively found in healthy subjects and fimA type II is most prevalent in periodontitis subjects [55, 56]. We detected salivary levels of total MMP-8, however Sorsa et al.’s [30, 31, 36, 37] results showed that, compared to total MMP-8, aMMP-8 was more effective at diagnosing periodontitis, so aMMP-8 may be a more valuable biomarker of periodontal diseases. To address these limitations, larger study sample size, longitudinal studies (such as experimental gingivitis or periodontitis designs), more precise biomarker selection, and more precise detection will be required to further evaluate our selected salivary biomarkers.

## Conclusion

Our study patient sample represented gingivitis and stage III periodontitis in the current classification of periodontal diseases, and our results showed that there were significant differences of salivary IL-1β, MMP-8, ICTP, and Pg between the healthy group, gingivitis group, and stage III periodontitis group. The efficacy of several prediction panels for diagnosing gingivitis and stage III periodontitis were evaluated. We found that the combination of IL-1β, ICTP, and Pg can be used to discriminate stage III periodontitis subjects from healthy subjects and gingivitis subjects. Further, the combination of IL-1β and MMP-8 can be used to discriminate gingivitis patients from healthy subjects.

## Data Availability

The datasets generated during the current study are available from the corresponding author on reasonable request.

## Abbreviations

IL-1β: 1Interleukin (IL)-1β
MMP-8: matrix metalloproteinase-8
ICTP: pyridinoline cross-linked carboxyterminal telopeptide of type I collagen
Pg: *Porphyromonas gingivalis*
BOP: bleeding on probing
PI: plaque index
PD: probing depth
CAL: clinical attachment loss
ELISA: enzyme linked immunosorbent assay
qPCR: quantitative real time polymerase chain reaction
ROC: receiver operating characteristics
AUC: area under the curve
POC: point of care
